# Robotic-assisted transverse colectomy for transverse colon cancer in a centenarian: a case report

**DOI:** 10.1093/jscr/rjag321

**Published:** 2026-04-28

**Authors:** Yoshinori Iwata, Chihiro Tanaka, Hironobu Takeuchi, Sho Miyazaki, Tatsuki Kawahara, Kazuo Yamamoto, Shinya Ohno, Shunya Kiriyama, Kakeru Tawada, Tomonari Suetsugu, Shuji Komori, Narutoshi Nagao, Masaki Katayama, Masahiko Kawai

**Affiliations:** Department of Surgery, Gifu Prefectural General Medical Center, 4-6-1 Noishiki, Gifu, Gifu 500-8717, Japan; Department of Surgery, Gifu Prefectural General Medical Center, 4-6-1 Noishiki, Gifu, Gifu 500-8717, Japan; Department of Surgery, Gifu Prefectural General Medical Center, 4-6-1 Noishiki, Gifu, Gifu 500-8717, Japan; Department of Surgery, Gifu Prefectural General Medical Center, 4-6-1 Noishiki, Gifu, Gifu 500-8717, Japan; Department of Surgery, Gifu Prefectural General Medical Center, 4-6-1 Noishiki, Gifu, Gifu 500-8717, Japan; Department of Surgery, Gifu Prefectural General Medical Center, 4-6-1 Noishiki, Gifu, Gifu 500-8717, Japan; Department of Surgery, Gifu Prefectural General Medical Center, 4-6-1 Noishiki, Gifu, Gifu 500-8717, Japan; Department of Surgery, Gifu Prefectural General Medical Center, 4-6-1 Noishiki, Gifu, Gifu 500-8717, Japan; Department of Surgery, Gifu Prefectural General Medical Center, 4-6-1 Noishiki, Gifu, Gifu 500-8717, Japan; Department of Surgery, Gifu Prefectural General Medical Center, 4-6-1 Noishiki, Gifu, Gifu 500-8717, Japan; Department of Surgery, Gifu Prefectural General Medical Center, 4-6-1 Noishiki, Gifu, Gifu 500-8717, Japan; Department of Surgery, Gifu Prefectural General Medical Center, 4-6-1 Noishiki, Gifu, Gifu 500-8717, Japan; Department of Pathology, Gifu Prefectural General Medical Center, 4-6-1 Noishiki, Gifu, Gifu 500-8717, Japan; Department of Surgery, Gifu Prefectural General Medical Center, 4-6-1 Noishiki, Gifu, Gifu 500-8717, Japan

**Keywords:** centenarian, transverse colon cancer, robotic-assisted surgery, intracorporeal anastomosis, clinical frailty scale, MSI-high

## Abstract

A 100-year-old woman with transverse colon cancer underwent robotic-assisted colectomy with intracorporeal anastomosis and Pfannenstiel specimen extraction. She was independent in activities of daily living and classified as non-frail based on a comprehensive geriatric assessment. Preoperative imaging showed localized disease without metastasis. The operation was completed without complications, and postoperative recovery was uneventful, with preservation of functional status. Histopathology revealed poorly differentiated adenocarcinoma with direct invasion into the jejunum and microsatellite instability (MSI) -high phenotype. At 6 months after surgery, the patient remains recurrence-free and ambulatory. This case suggests that curative minimally invasive surgery may be feasible in carefully selected centenarian patients. Functional assessment rather than chronological age alone should guide surgical decision-making in super-elderly individuals.

## Introduction

With increasing life expectancy, surgeons are encountering more super-elderly patients with colorectal cancer [1]. Surgical indications in centenarians remain controversial because of limited physiological reserve and concerns about postoperative functional decline [2]. Evidence specifically addressing surgical management in patients aged 100 years or older is extremely limited [1]. Minimally invasive surgery may reduce surgical stress and facilitate recovery, but reports in centenarian patients are rare [3–6]. Minimally invasive surgery may be particularly advantageous in centenarian patients because reduced surgical trauma may help preserve postoperative functional independence. We present a case of transverse colon cancer successfully treated with robotic-assisted colectomy in a 100-year-old woman, focusing on functional patient selection and short-term outcomes.

## Case presentation

**Figure 1:**
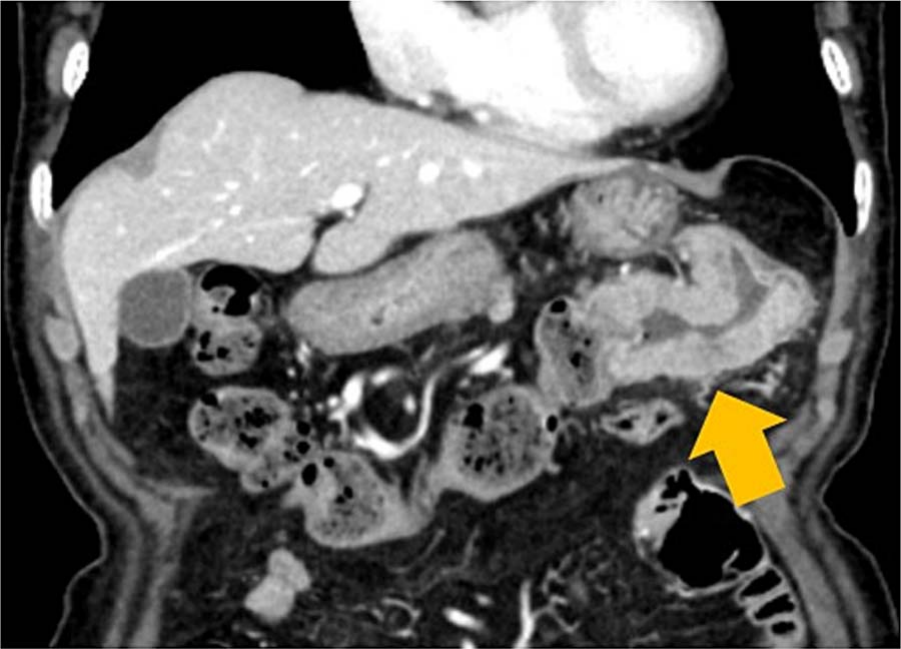
Contrast-enhanced CT findings. Axial view showing a 60-mm mass at the splenic flexure without evidence of distant metastasis.

A 100-year-old woman was referred for evaluation of anemia. Colonoscopy revealed an ulcerated lesion in the transverse colon, and biopsy confirmed adenocarcinoma. Contrast-enhanced computed tomography (CT) demonstrated a localized tumor without distant metastasis (Fig. 1).

The patient lived independently, had no cognitive impairment, and had only well-controlled hypertension. Comprehensive geriatric assessment classified her as non-frail with a Clinical Frailty Scale score of 3, which corresponds to a patient who is managing well with preserved independence in daily activities [7]. After discussion, the patient and her family requested definitive surgical treatment.

**Figure 2:**
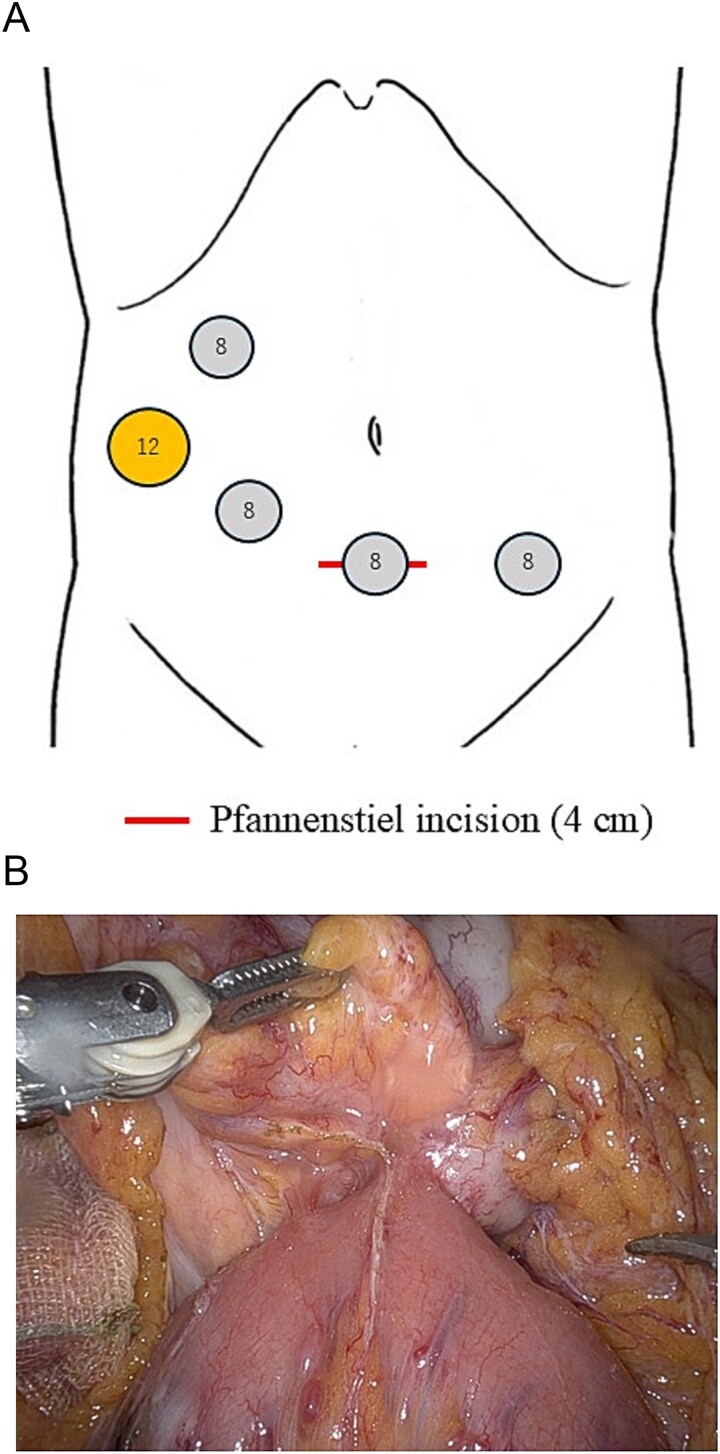
(A) Port placement for robotic-assisted transverse colectomy. Four 8-mm robotic ports and one 12-mm assistant port were used. The specimen was extracted through a 4-cm Pfannenstiel incision. (B) Intraoperative findings. The tumor at the splenic flexure showed direct invasion into the proximal jejunum, requiring partial jejunal resection.

**Figure 3:**
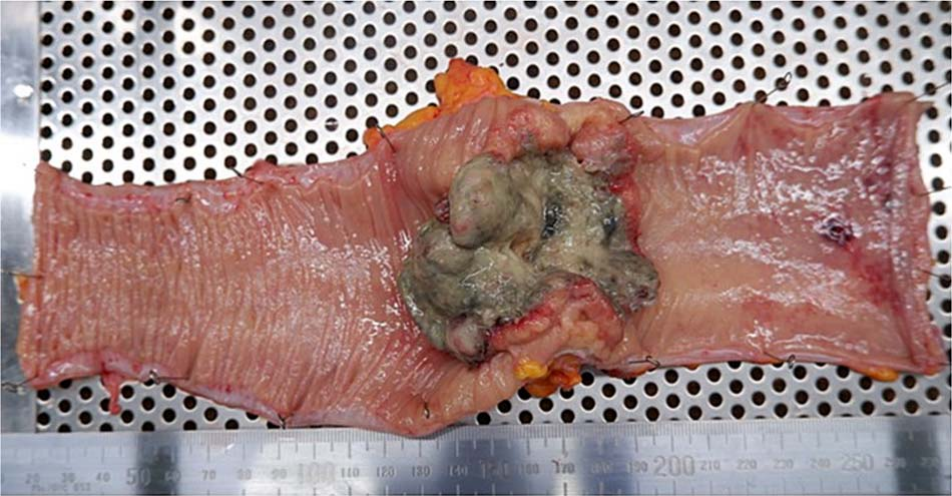
Gross specimen showing a circumferential tumor invading the jejunum.

Robotic-assisted transverse colectomy with intracorporeal anastomosis was performed. Four 8-mm robotic ports and one 12-mm assistant port were placed (Fig. 2A). The specimen was extracted through a 4-cm Pfannenstiel incision to minimize postoperative pain. Partial jejunal resection was added because of suspected direct invasion (Fig. 2B). Operative time was 198 min with minimal blood loss. Gross specimen showing a circumferential tumor invading the jejunum (Fig. 3).

The postoperative course was uneventful. Early ambulation and oral intake were achieved without complications, and the patient was discharged home on postoperative Day 13 with preserved independence.

**Figure 4:**
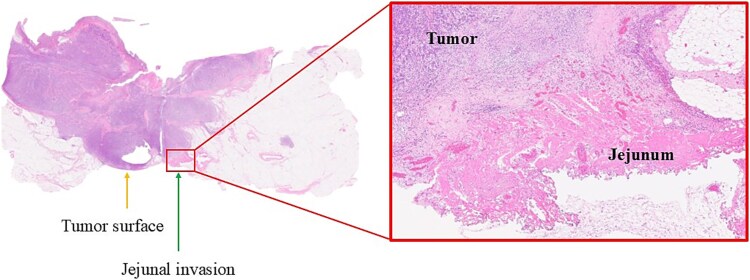
Histopathological findings. Hematoxylin–eosin staining demonstrating poorly differentiated adenocarcinoma with invasion into the jejunum.

Histopathology revealed poorly differentiated adenocarcinoma with direct invasion into the jejunum (pT4b) and metastasis in one regional lymph node (pN1a), corresponding to stage IIIB disease (Fig. 4). Immunohistochemistry showed loss of MLH1 and PMS2 expression, indicating mismatch repair deficiency and MSI-high status. BRAF V600E was negative. At 6 months, the patient remains recurrence-free and ambulatory.

## Discussion

Management of colorectal cancer in centenarians is challenging because of frailty, comorbidities, and limited physiological reserve [2]. However, chronological age alone should not preclude curative treatment when functional status is preserved [7, 8]. In this case, independence in activities of daily living, non-frail status, and absence of major comorbidities supported the surgical indication [7].

Minimally invasive surgery has been reported to reduce postoperative pain, attenuate the systemic inflammatory response, and promote earlier postoperative recovery compared with conventional open surgery [3–6]. These advantages may be particularly relevant in super-elderly patients with limited physiological reserve. In addition, avoidance of a midline laparotomy incision may reduce the risk of adhesive small bowel obstruction and incisional hernia. Intracorporeal anastomosis combined with Pfannenstiel specimen extraction may further minimize wound-related complications and postoperative discomfort [9, 10].

The MSI-high phenotype is more common in elderly patients [11] and may be associated with favorable prognosis when curative resection is achieved [12]. Our institutional analysis also demonstrated favorable outcomes in MSI-high colorectal cancer [13]. Although immune checkpoint inhibitors are emerging treatments for MSI-high tumors [14, 15], surgery remains the only established curative option for localized colon cancer [12].

This report is limited by its single-case nature and short follow-up period. Nevertheless, it demonstrates that curative colorectal surgery can be safely performed in carefully selected centenarian patients.

## Conclusion

Robotic-assisted transverse colectomy combined with intracorporeal anastomosis and Pfannenstiel specimen extraction was safely performed in a carefully selected centenarian patient. Functional and frailty-based assessment rather than chronological age alone should guide surgical decision-making. An appropriate minimally invasive strategy may help minimize surgical trauma and postoperative pain, contributing to favorable recovery and preservation of functional status in super-elderly individuals, even as non-surgical treatment options continue to evolve. Avoidance of a midline laparotomy incision may also reduce wound-related complications such as incisional hernia and adhesive bowel obstruction, which may be particularly beneficial in super-elderly patients.
